# Beyond Research Priorities: Stakeholder‐Identified Health System Challenges From an Australian Childhood Cancer Priority‐Setting Partnership

**DOI:** 10.1111/hex.70884

**Published:** 2026-09-27

**Authors:** Gayani De Silva, Eden G. Robertson, Alison Bowers, Jason D. Pole, Sheila K. Patel, Joanne Cummings, Natalie Bradford

**Affiliations:** ^1^ Cancer & Palliative Care Outcomes Centre, Centre for Healthcare Transformation, Faculty of Health Queensland University of Technology Brisbane Queensland Australia; ^2^ Discipline of Paediatrics and Child Health, School of Clinical Medicine UNSW Medicine & Health, Randwick Clinical Campus, UNSW Sydney, Randwick New South Wales Australia; ^3^ Behavioural Sciences Unit Kids Cancer Centre, Sydney Children's Hospital Randwick New South Wales Australia; ^4^ Queensland Digital Health Centre, Faculty of Health, Medicine and Behavioural Sciences University of Queensland Brisbane Queensland Australia; ^5^ Parent Representative on the Child and Adolescent Cancer Priority Setting Partnership Melbourne Victoria Australia; ^6^ Department of Medicine University of Melbourne Melbourne Victoria Australia; ^7^ Anglicare New South Wales Australia; ^8^ Viertel Cancer Research Centre, Cancer Council Queensland Brisbane Queensland Australia

**Keywords:** childhood cancer, health system, policy‐relevant concerns

## Abstract

**Background:**

Priority‐setting partnerships (PSPs) are used to identify research questions that matter most to patients, caregivers, and clinicians. However, submissions that fall outside the scope of research prioritisation are rarely examined despite their potential to reveal important unmet needs. This study explored concerns relevant to practice and policy using the out‐of‐scope questions submitted during the Australian Child and Adolescent Cancer PSP.

**Methods:**

We conducted the Australian Child and Adolescent Cancer PSP with the James Lind Alliance to identify research priorities for childhood cancer. An online national survey collected submissions from children and adolescents diagnosed with cancer, their caregivers, and healthcare professionals. We identified submissions that fell outside the PSP scope. These were collated and analysed using qualitative content analysis, followed by framework analysis to deductively map themes to the World Health Organisation Health System Building Blocks, enabling system‐level interpretation of community‐identified concerns.

**Results:**

Of the 701 submissions, 99 (14%) were classified as out‐of‐scope and included in this analysis. Seventy‐two respondents contributed to these submissions, most of whom were caregivers (n = 58, 81%). Themes mapped across the WHO building blocks, including treatment access, research innovation and translation, health financing, service delivery and environment of care, leadership and governance, health information and communication, and health workforce. Additional concerns were classified as dimensions of care.

**Conclusions:**

Out‐of‐scope PSP submissions provide valuable insights into patient, caregiver, and clinician concerns that complement research priorities. They highlight persistent challenges in access to care, supportive services, workforce capacity, information provision, and financial burden, underscoring that many family concerns stem from gaps in implementation, service delivery, and policy, rather than evidence alone. These system‐level challenges must be communicated to funders, policymakers, charities, and consumers to translate these insights into meaningful improvements in care.

## 1 Introduction

Priority setting partnerships (PSPs) provide a systematic approach to identify the most pressing unanswered questions to inform a focused research agenda in a given area [1]. Increasingly, early involvement of patients, survivors, and caregivers in such approaches are recognised as essential to ensuring relevance, equity, and maximising the impact of resulting research [2]. Many research prioritisation exercises have been undertaken across a range of health disciplines, including initiatives focused on identifying research priorities for different health areas and in diverse contexts [3, 4, 5]. These exercises typically define a specific project scope and focus on researchable questions characterised by verified uncertainties in the existing evidence base [6]. However, a substantial proportion of submissions to PSPs are categorised as out‐of‐scope, because they do not align with the PSP's defined remit or meet the criteria for representing unanswered, researchable questions. Often, these submissions articulate broader community concerns that may not be captured within the boundaries of the final research priorities [7]. This is despite reflecting issues that stakeholders experience as urgent, unresolved, and impactful in their everyday interactions with health systems.

PSPs that set aside out‐of‐scope questions once research priorities are established risk the loss of stakeholder‐identified concerns relevant to service design, policy development, and quality improvement [8, 9]. In many PSPs, such excluded submissions are neither publicly reported nor subjected to further analysis, limiting transparency and the broader utility of participant contributions, except in limited circumstances [10, 11]. Although these submissions fall outside the formal scope of defined research prioritisation, they represent an important source of information that provokes system‐level actions, informs health service reforms, policy changes, or improved communication and knowledge translations which may not be fully achieved through research alone. Identifying and analysing out‐of‐scope submissions separately permits a clear distinction between research priorities and broader system‐ and policy‐level needs [12]. Examining these submissions offers valuable insight into gaps that may be overlooked in traditional research prioritisation processes [8]. It also provides a complementary perspective on stakeholder concerns, revealing areas where policy reform, service redesign, or improved knowledge translation may have greater impact than research alone [9]. Importantly, this approach helps define the boundary between researchable uncertainty and implementation challenges and shows how health system structures influence patient and provider experiences.

The Australian Child and Adolescent Cancer Research PSP [13] was established using the James Lind Alliance (JLA) methodology [7] to identify shared research priorities from the perspectives of patients, caregivers, and health professionals. The scope of this PSP was defined and agreed by the PSP steering group, which included childhood cancer survivors and their caregivers with lived experience, as well as health professionals providing childhood cancer care. The scope encompassed any type of cancer, including brain tumours, diagnosed between birth and 19 years of age, addressing questions across the full cancer continuum. It included answerable research questions related to prevention, screening, diagnosis, and treatment through end‐of‐treatment care, relapse, survivorship, late effects, and palliative and end‐of‐life care, while also considering the physical, psychological, emotional, social, financial, and spiritual aspects of cancer affecting children and adolescents along the care pathway [13]. This paper explores the out‐of‐scope submissions generated during this process. We aimed to characterise the nature of the out‐of‐scope submissions and situate them within a health systems and policy context. By doing so, we seek to demonstrate how submissions excluded from research prioritisation can nevertheless offer valuable guidance for policy reform, service improvement, and more responsive childhood cancer care systems.

## 2 Methods

### 2.1 Recruitment and Sample

Between August and November 2024, we invited eligible participants to submit questions relating to issues they perceived as unanswered by research in the context of child and adolescent cancer via an online national survey administered through Qualtrics. Eligible participants included children and adolescents diagnosed with any type of cancer or brain tumour before their 19th birthday, their caregivers, and healthcare professionals involved in providing care to the childhood cancer community. Participation was voluntary, and responses were submitted anonymously. Details on the recruitment strategy and survey have previously been published, and the overall process was overseen by a national steering group comprising people with lived experience and clinicians [14].

### 2.2 Data Collection

As part of the structured PSP process, submissions were initially screened by GD against the pre‐defined scope agreed by the project team (ER, JP, JC, NB, SP). Submissions that did not align with the predefined PSP scope were collated separately and retained for secondary analysis. The team reviewed the full list of submissions to identify any additional out‐of‐scope items and to confirm initial classifications. This subgroup reviewed each submission in detail and reached consensus on all submissions deemed out of scope. The full PSP steering group then reviewed and confirmed all submissions deemed out of scope.

### 2.3 Analysis

Out‐of‐scope submission analysis was conducted in two stages. First, an inductive qualitative content analysis was undertaken to systematically examine the issues and concerns expressed. Three researchers (GD, NB, SP) independently reviewed each submission and coded it into categories based on its primary focus. Through an iterative process of comparison and discussion, submissions were grouped into seven analytically derived domains: service delivery and environment of care; health workforce; health information and communication; treatment access and research innovation and translation; health financing; leadership and governance; and dimensions of care. This inductive phase allowed important system‐level concerns to emerge directly from respondents’ accounts, without imposing a priori assumptions about their relevance and categorical placement [15].

Following inductive categorisation, we observed that the identified domains aligned closely with the World Health Organization's (WHO) Health System Building Blocks framework: service delivery, health workforce, health information systems, access to essential medicines, financing, and leadership/governance [16]. We therefore conducted a subsequent deductive mapping, whereby each emergent domain was assigned to the most relevant WHO health system building block according to its primary focus. The framework was not used to generate the initial categories; rather, it served as a secondary analytical lens to support systems‐level interpretation of the findings. This framework represents the core components required for effective and efficient health system functioning. It enables a structured systems‐level analysis, allowing community concerns to be mapped to areas of systemic strengths and weaknesses. This approach allowed individual concerns to be interpreted within a broader health systems context and facilitated consideration of implications for service delivery, policy, governance, workforce capacity, financing, and access to care [17]. Where submissions intersected multiple system components, they were mapped to the most relevant building block based on their dominant focus. This framework analysis also facilitated identification of cross‐cutting and interconnected issues across services, workforce, policy, and financing, highlighting areas where system‐level reform may be required [18].

We categorised out‐of‐scope submissions into a seventh domain to capture broader concerns, values, and reflections beyond conventional research questions, particularly those that could not be readily aligned with the WHO framework. These included submissions addressing philosophical, ethical, or experiential dimensions of care rather than discrete system components. By structuring them in this way, we ensured that all out‐of‐scope concerns were systematically examined through a policy and service reform lens, even when they fell outside the formal remit of research prioritisation. To acknowledge and represent this distinct dimension, these contributions were collectively conceptualised as the “dimensions of care.”

Findings are presented in narrative form with selected exemplars to represent the data and relevant domains. Identifiers are removed to maintain confidentiality.

## 3 Results

Among 701 total submissions from 229 participants, 99 submissions were identified as out‐of‐scope. These submissions came from 72 respondents: 58 (81%) caregivers, 10 (14%) health professionals and four (5%) survivors. Caregivers contributed the most out‐of‐scope submissions across all 7 domains: 71 (71.7%) of 99.

The findings are presented below according to the domains identified through qualitative content analysis and subsequently interpreted using the WHO Health System Building Blocks framework. These are presented as verbatim quotes from respondents, without editing for typographic errors. The largest proportions of concerns were related to the domains of treatment access, research innovation, and translation (n = 26, 26%), followed by health financing (n = 19, 19%) (Table 1).

**Table 1:** Distribution of out‐of‐scope submissions by respondent type and domain (*n* = 99).

Domains	Caregivers *n* (%)	Health professionals *n* (%)	Patients/survivors *n* (%)	Total *n* (%)
1. Service delivery and environment of care	9 (13)	3 (14)	1 (14)	13 (13)
2. Health workforce	3 (4)	0 (0)	1 (14)	4 (4)
3. Health information and communication	8 (11)	1 (4)	2 (28)	11 (11)
4. Treatment access, research innovation, and translation	20 (28)	5 (23)	1 (14)	26 (26)
5. Health financing	14 (19)	4 (19)	1 (14)	19 (19)
6. Leadership and governance	9 (13)	4 (19)	0 (0)	13 (13)
7. Dimensions of care	8 (11)	4 (19)	1 (14)	13 (13)
Total number of concerns	71	21	7	99

### 3.1 Service Delivery and Environment of Care

We received 13 submissions (13%) mapped to this domain. Several respondents questioned the availability of age‐appropriate, supportive environments in hospitals, emphasising the importance of privacy and dignity during treatment.

Why aren't they separating teenagers from younger children in hospitals?

(caregiver)

How can we find ways for cancer patients to be in their own rooms during treatments. Should not have to be sharing rooms with other families during this difficult time.

(caregiver)

Respondents further described the service gaps in the emotional and logistical demands of cancer care, with families questioning:

Every support organisation from hospitals to not for profits claim to be client‐centred and strengths based. What measures can be used to identify whether organisations are achieving that, and the areas in which they need to improve in order to achieve it?.

(health care professional (HCP))

Why isn't there a social care for kids and parents on the ward?/Why isn't there a social and risk factors index or policy list a priority?

(caregiver)

### 3.2 Health Workforce

A few respondents raised concerns about workforce education, retention and appropriate recognition of the highly skilled professionals in childhood cancer care:

As a parent of a [child] who underwent a bone marrow transplant, I would like to see the nurses in that area be paid more. They are so specialised in their expertise and the medications they are dealing with are very complex. To retain these nurses, they should receive extra payment.

(caregiver)

Can education institutions do more to create oncology pathways for health professionals to ensure there are highly skilled clinicians equipped with the latest research and treatment methods for childhood cancer?

(survivor)

### 3.3 Health Information and Communication

Respondents described multiple challenges related to access, clarity, and consistency of health information and communication across the childhood cancer care continuum. They reported inadequate or unclear communication at the time of diagnosis, describing difficulties understanding the nature and seriousness of the disease, treatment intent, and urgency of care:

I had no idea how serious my leukaemia was. I was never told what kind it was.

(survivor)

How long do I have to get [treatment at] the hospital?

(survivor)

They perceived the need for information on their fundamental questions about the disease and management: aspects of treatment decisions, symptom management, and late effects, suggesting ongoing uncertainty and limited shared understanding during and after treatment:

How does the body experience pain and what are the ways that it can be treated?

(caregiver)

Why do 1 year old [children] get the same dose of chemotherapy as adults?

(survivor)

### 3.4 Treatment Access and Research Innovation and Translation

Respondents frequently highlighted limited access to essential treatments, procedures, and services, with comparisons drawn with models in other countries:

Are all worldwide treatment options available in Australia. Ie why do some families need to travel to the US for medication and treatment?

(caregiver)

In USA paediatric cancer patients often travel interstate to receive specialist care – should we be setting up centres of excellence in Australia for rarer types of cancer such as types of sarcoma? Would this result in better outcomes?

(caregiver)

Respondents also highlighted missed opportunities for system efficiencies:

What can be done at a system level to ensure cancer patients receive treatment when they need it, and not when hospital policy allows. i.e., insertion or removal of Hickman/ports only being done when designated hospital lists allow, resulting in delays of effective treatment or delay in removal following the need to have one.

(caregiver)

How do we use technology to our advantage to streamline processes, rather than making them more complex every time?

(HCP)

Some noted concerns about the perceived rigidity of treatment protocols and regulatory oversight, particularly where protocols limit individualised treatment decisions:

It would have been better if we considered options outside of chemo. For brain tumours where chemo often doesn't work.

(caregiver)

Is it possible for treatment to be individualised by choice of the parent/treating team? The system seemed to be that once categorised at diagnosis that you would not be acting in the best interest of the child for declining parts of the treatment protocol.

(caregiver)

Respondents consistently identified a substantial gap between research discovery and clinical implementation:

One of the things I will always wonder about is how much of what is learned in practice is fed back and applied as a learning to all departments, especially back to the research areas.

(caregiver)

I have heard cancer treatment of my child's specific cancer has progressed in the research stage but is way behind in implementation due to fund[ing] and bureaucracy. Why is this so?

(caregiver)

### 3.5 Health Financing

Respondents raised concerns about insufficient investment in childhood cancer treatment and resources:

It would be wonderful if more money was poured into development of new treatments for childhood cancer.

(caregiver)

One of the things I wonder about is what incentives can we offer or legislative requirements can we put in place to ensure drug companies develop drugs for paediatric cancers.

(HCP)

They consistently described the financial burden placed on families, particularly in relation to long‐term treatment‐related costs: “Why are corporations such as insurance companies allowed to discriminate against my child who has finished treatment and is regarded as cured but now has to pay higher [insurance] premiums?”

(caregiver)

Are survivors entitled to concessions or rebates for the costs of medications and/or treatment of chronic long term side effects or conditions?

(survivor)

Some expressed the distress that families felt compelled to fundraise to access investigational treatments, raising concerns about equity for families without financial or social capital:

One of the things I wondered about was if the child's family wasn't in a position to fund raise or have generational wealth, did they just die? That also kept me awake at night. Why doesn't the government help finance the overseas treatment?

(caregiver)

We shouldn't have to rely on charities to support kids especially post treatment. What more can be done?

(caregiver)

### 3.6 Leadership and Governance

Respondents consistently expressed a desire for greater inclusion of caregivers, patients, and survivors in governance, research prioritisation, and service design. Many described feeling unheard or excluded from decision‐making processes:

Can more opportunities be created for parents of children, or the children themselves where appropriate, to be involved in research and the development of future focus of treatment? Including advisory groups, board, governance.

(caregiver)

I would like to see a directory of studies and or surveys such as this from a global network asking parents and survivors in depth specific questions on a number of topics to hone the types of answers we are all searching for or may be interested in the long term and for research and development purposes.

(caregiver)

They further expressed concerns about limited parental autonomy in challenging treatment protocols once initiated:

When can parents challenge treatment paths of medical teams?

(caregiver)

Why do we sign our kids up for a treatment protocol which is hard to stray from once you have started without having done all investigative scans and tests?

(caregiver)

### 3.7 Dimensions of Care

There were a few submissions considered as conceptual concerns and reflections that were excluded. These included broader aspects of care:

AYA cancer care (caregiver), “Diagnoses and treatments in the future” (caregiver), Psychosocial care”(caregiver), “Early survivorship care and models of survivorship care”(HCP), “Pain management and novel therapies.”

(survivor)

Families described their emotionally driven lived experience of difficulty in building trust with clinical teams and navigating hospital systems:

For me getting to know Dr and the nursing staff was the hardest part. Once that relationship is built and knowledge of the hospital together, everything is easier.

(caregiver)

Others expressed gratitude for experiencing successful life‐saving interventions:

What really mattered to me was that my 19 month old survived neuroblastoma.

(caregiver)

I am so grateful that the doctors saved my daughter's life.

(caregiver)

The complete data with all out‐of‐scope submissions are available in Supplemental Material 1.

## 4 Discussion

Out‐of‐scope submissions represented 14% of the 701 Australian Childhood Cancer PSP survey submissions and provide valuable insight into the wider realities, frustrations and hopes of survivors, caregivers and health professionals. Caregivers were the most represented group, reflecting their strong engagement in the process and the breadth of their practical, emotional, and system‐level concerns that extend beyond research‐focused questions. This underscores the deep interdependence between improved survivor outcomes and family well‑being, aligning with evidence that childhood cancer imposes long‐term, whole‐of‐family impacts and requires coordinated, family‐centred care models [19].

The findings are discussed across the key domains of the WHO Health System Building Blocks framework, with particular consideration of their implications for service delivery, health workforce capacity, access to treatments and services, health financing, information provision, and system governance. This mapping revealed interconnected challenges across different areas of the health system, highlighting the need for integrated policy responses and emphasising structural and organisational determinants of care quality and access in complex, long‐term health conditions [17]. From a policy standpoint, these findings suggest that PSPs may serve a dual purpose: informing research agendas while also generating actionable information for people‐centred health system improvement [20].

Findings indicate that the physical and social environments of childhood cancer care may be inconsistently aligned with the developmental and psychosocial needs of children and adolescents. This is consistent with prior research highlighting the importance of comprehensive physical and emotional support infrastructure, particularly in settings with high levels of unmet need [21, 22]. The limited availability of services that extend beyond acute clinical care, including dedicated social spaces, psychosocial supports, and palliative or hospice services, was evident, reflecting persistent gaps that remain widely reported in paediatric oncology settings globally [23, 24]. Collectively, they point to the need for system‐level reforms, including child‐ and youth‐centred planning, the establishment of minimum standards for supportive care environments, and alignment with international guidelines and networks that promote evidence‐based supportive care [25]. Incorporating measurable indicators of family experience and accountability may further support the consistent translation of commitments to client‐centred care in routine practice.

Concerns regarding workforce resourcing and retention were also identified, as access to high‐quality cancer care depends on a well‐supported workforce [26]. These findings align with national workforce priorities articulated in the Australian Cancer Plan, emphasising a resilient, future‐ready cancer care workforce [27]. Building the capacity of multidisciplinary teams to address these issues should be supported.

Ongoing concerns regarding access to novel therapies, specialist expertise, and clinical trials reflect perceived inequities in national coordination and delays in the timely adoption and availability of emerging therapeutics. Evidence indicates that such inequities disproportionately affect Aboriginal and Torres Strait Islander children and families, culturally and linguistically diverse populations, and those residing outside metropolitan centres, who continue to experience poorer survival outcomes and reduced access to culturally safe follow‐up care [28, 29]. Concerns were also evident between the delivery of standardised, protocol‐driven care and the need for greater individualisation based on tumour biology, treatment response, and family preferences [30]. While standardised care remains essential for safety and quality, equitable access to appropriate treatment options remains central to patient‐centred paediatric oncology care [31].

Submissions further pointed to gaps between research discovery and clinical implementation, reinforcing the policy importance of sustained investment in translational infrastructure and coordinated clinical trial networks. Despite national initiatives aimed at improving equitable access to precision oncology [32], families perceived uneven progress, particularly in trial availability, age group inclusion, and the timely adoption of innovative care models, for adolescents and young adults and those living outside major centres. Important next steps should be aimed at policy directions, concentrating expertise, developing clinical trial‐ready infrastructures and reducing the burden of travel for families [33, 34] while enabling culturally safe collaboration nationally and internationally through harmonised and equitable participation in clinical trials [35, 36].

Financial concerns also featured prominently, reflecting dissatisfaction with fragmented funding arrangements and short funding cycles that inadequately support long‐term care and survivorship. Financial burden was perceived as ongoing and extending well beyond active treatment, consistent with evidence showing persistent healthcare utilisation and out‐of‐pocket costs into survivorship [37]. Limited insurance coverage for late‐effects monitoring and supportive care further underscores the vulnerability of survivors across the life course [38, 39]. Policy responses that strengthen long‐term financial protection and clarify survivorship entitlements may therefore be more impactful than further research alone.

Access to information and communication about the disease and available services emerged as a cross‐cutting concern, underpinning many of the issues raised across submissions. A considerable proportion of out‐of‐scope responses reflected needs that could potentially be addressed through better dissemination of existing evidence, clearer communication strategies, and more consistent implementation of established clinical and supportive care standards, rather than through the generation of new research. Families must navigate complex and fragmented health systems with uneven access to survivorship care, psychosocial support, and long‐term monitoring, making timely and appropriate information critical. The challenge lies less in the availability of information than in a mismatch between what families need and how information is delivered [40]. Respondents emphasised the need for accessible, plain‐language information about care pathways, service availability, and the translation of research into practice. These findings are consistent with evidence highlighting the importance of clear communication and shared decision‐making in paediatric oncology, where parents and adolescents often report unmet information needs and uncertainty when navigating complex treatment decisions [41]. Interventions such as decision aids have shown support in facilitating informed choices, promoting shared decision‐making, and enhancing engagement in care planning [42]. These findings also align with policy efforts to enhance transparency, coordination, and consumer engagement through digital health tools and co‐designed information resources. Findings highlight gaps in meaningful patient and family involvement in paediatric cancer care and research, with perceptions of exclusion and limited transparency indicating misalignment with established consumer engagement standards [43]. International best‐practice frameworks increasingly recognise caregivers and young people as partners at the policy and system‐level in governance, research prioritisation, and service design, particularly those that need high‐stakes and protocol‐driven care, such as in paediatric oncology [44].

Although out‐of‐scope submissions did not meet PSP inclusion criteria to propose as future research questions, they consistently highlighted system‐level shortcomings that explained patient and family experiences. Analysed systematically, these submissions offer a valuable lens on where policy reform, service redesign, or improved knowledge translation may yield greater benefit, in addition to prioritised research. Out‐of‐scope submissions provide a powerful reminder that care is shaped not only by research priorities but by the policies, systems, and communication practices that determine whether families can access, understand, and benefit from available care.

### 4.1 Strengths and Limitations

This analysis captures policy‐ and service‐relevant insights that extend beyond proposing research priorities and provides a more comprehensive understanding of stakeholder concerns across the childhood cancer care continuum. Mapping submissions to the WHO health system building blocks offers a conceptual framework for interpreting system‐level issues, facilitating translation of stakeholder input into health services and policy‐relevant findings. This deductive mapping to a predefined framework after an inductive categorisation approach aligns with recent guidance advocating the combined use of inductive and deductive logic in qualitative health research to balance data‐driven insight with theory‐informed interpretation. The inclusion of diverse perspectives and its national scope further strengthen the relevance of this analysis for informing service reform. However, several limitations should be acknowledged. First, these out‐of‐scope submissions were not originally designed to elicit detailed policy or service feedback, limiting the depth and specificity of some responses and precluding formal assessment of the feasibility or impact of proposed solutions by respondents. Still, they represent real concerns of those with lived experience. Also, submissions may over‐represent motivated caregivers with higher health literacy and under‐represent groups with limited capacity to engage, including culturally and linguistically diverse communities. Second, classification of submissions as out of scope and their subsequent thematic mapping involved interpretive judgement, introducing potential subjectivity. Finally, while the findings highlight important system‑level issues, the analysis does not quantify their relative priority, nor does it assess the effectiveness of specific policy or service interventions. Further work is required to determine the relative priority and to assess the effectiveness of specific policy or service interventions.

### 4.2 Implications

While prioritising research uncertainties is essential to identifying unmet research needs in childhood cancer, it is equally important to strengthen the policies, services, and communication systems that support the effective implementation of research outcomes and contribute to comprehensive improvements in childhood cancer care. It is important for health systems to clearly distinguish researchable uncertainties and implementation failures, filtering and directing system‐level concerns toward appropriate policy, governance, or service redesign mechanisms rather than research pipelines alone. Health services and policymakers should systematically review and act on these submissions, alongside research priorities, to ensure that stakeholder concerns translate into tangible improvements in care delivery, service design, and health system performance.

The results highlight the need for more integrated, family‐centred models of childhood cancer care through routine attention to address gaps in communication, supportive care and survivorship across the care continuum. Particular attention should be given to caregiver psychosocial, informational, practical, and financial needs, recognising the central role of families in childhood cancer care. Strengthening supportive care infrastructure, including child‐ and youth‐friendly environments, psychosocial services, palliative care, and survivorship programs, alongside investment in a well‐supported multidisciplinary workforce, may improve both care quality and patient experience. Health systems should prioritise stronger coordination between care settings, equitable access to specialist expertise, novel therapies, and clinical trials, and mechanisms for meaningful patient and family involvement in service design and decision‐making.

Policy initiatives should also consider opportunities to streamline and sustain regulatory pathways and strengthen cross‐border collaboration, helping ensure that advances in childhood cancer care translate into equitable global benefits. In parallel, policy reform should prioritise outcome‐linked funding models, financial protections for survivors and caregivers, and targeted investment in areas with weak commercial incentives, including rare cancers and child‐specific drug development.

## 5 Conclusion

The Australian Child and Adolescent Cancer Research PSP ensured that out‐of‐scope questions were documented and analysed, ensuring that concerns based on the lived experience were never lost. Analysis and mapping using the WHO Health System Building Blocks framework indicates that families and clinicians perceive persistent, interconnected system‐level challenges spanning health service delivery, funding arrangements, research structures, information pathways, and governance across the cancer care continuum. By applying a health systems lens to stakeholder concerns, this provides valuable insights for health system improvement alongside research agenda setting. Future PSPs should routinely consider analysing such submissions as a complementary source of information for health system improvement, while policymakers and health services should use these insights to inform service redesign, implementation, and equitable, family‐centred childhood cancer care. Importantly, these insights bridge research prioritisation and health‐system improvement.

## Author Contributions

**Gayani De Silva:** conceptualisation, methodology, investigation, data curation, formal analysis, visualisation, project administration, writing – original draft, writing – review and editing. **Eden G. Robertson:** methodology, supervision, formal analysis, writing – review and editing. **Alison Bowers:** methodology, supervision, formal analysis, writing – review and editing. **Jason D. Pole:** methodology, writing – review and editing, formal analysis. **Sheila K. Patel:** methodology, investigation, formal analysis, writing – review and editing. **Joanne Cummings:** methodology, writing – review and editing, formal analysis. **Natalie Bradford:** conceptualisation, supervision, funding acquisition, writing – review and editing, methodology, formal analysis, investigation. All the authors listed above have contributed substantially to the manuscript and have agreed to the final submitted version.

## Ethics Statement

This study involves the analysis of secondary data collected from human participants. We received ethics approval from the Children's Hospitals Network Human Research Ethics Committee (HREC/2024/QCHQ/104116) and adhered to the tenets of the Declaration of Helsinki. We obtained governance approval to collect data from patients and families at Queensland Children's Hospital from Queensland University of Technology – Health Translation Queensland, and Children's Hospital Queensland and secured administrative approval from Queensland University of Technology to carry out the study as part of a PhD project. Participants gave informed consent to participate in the study and to publicise their research uncertainties before taking part. The main Priority Setting Partnership study followed standard ethical and engagement practices used by JLA PSPs, including clear information on participation and data handling.

## Conflicts of Interest

The authors declare no conflicts of interest.

## Supporting information


Supporting File

## Data Availability

Data are available upon reasonable request from the corresponding author. The majority of data that support the findings of this study are openly available via the James Lind Alliance website at https://www.jla.nihr.ac.uk/priority-setting partnerships/child& adolescent‐cancer‐australia/.
