# Supplementary material for: Beyond Research Priorities: Stakeholder‐Identified Health System Challenges From an Australian Childhood Cancer Priority‐Setting Partnership
Source: Health Expect. 2026 Sep 27;29(5):e70884. doi: 10.1111/hex.70884 (PMC13617300; doi:10.1111/hex.70884)
Supplement: Supplementary file 1 [file HEX-29-e70884-s001.docx]

**Supplemental file 1:**

| **Main theme** | **Respondent** | **Deidentified Quote** |
| --- | --- | --- |
| Service delivery and environment of care | Caregiver | How can we find ways for cancer patients to be in their own rooms during treatments. Should not have to be sharing rooms with other families during this difficult time. |
|  | Caregiver | Why are children in hospitals given such bad food that they just put them on 'nutrition' which is way worse for their body than giving them real foods. |
|  | Caregiver | Why aren't they separating teenagers from younger children in hospital? |
|  | Caregiver | Why isn't there a social are for kids and parents on the ward?/Why isn’t there a social and risk factors index or policy list a priority? |
|  | Caregiver | Why isn't the social emotional wellbeing of cancer kids a priority? |
|  | Caregiver | It would have been better if there was a social worker who worked with us, not against us when our son was diagnosed. Our first social worker told us, a remote familywith 3 children 5 and under, that “Ronald McDonald House is not for people like you”; despite the fact we were over 1600km from home |
|  | Caregiver | Why isn’t brain cancer treated as an acquired brain injury at SCHW and why isn’t more rehab provided? |
|  | Health professional | How can we develop dedicated late‑effects/survivorship services with GP/shared‑care protocols and clear re‑entry pathways? |
|  | Health professional | Every support organisation from hospitals to not for profits claim to be client centered and strengths based. What measures can be used to identify whether organisations are achieving that, and the areas in which they need to improve in order to achieve it. |
|  | Caregiver | Why is there no paediatric hospice in South Australia? |
|  | Caregiver | Travel is sooo frustrating and complicated and with so much on our plate, 3 monthly trips back to Brisbane, haveing to also deal with that was too much. |
|  | Health professional | How do we ensure access to state of the art treatments? |
|  | Survivor | Are there any regional long term followup clinics? |
| Health workforce | Caregiver | As a parent of a good who underwent a bone marrow transplant I would like to see the nurses in that area be paid more. They are so specialised in their expertise and the medications they are dealing with are very complex. To retain these nurses they should receive extra payment. It cast be said that a nurse who is caring for someone with say a boring arm has to have the same skill level and expertise as a BMT nurse. |
|  | Caregiver | Can education institutions do more to create oncology pathways for health professionals to ensure there are highly skilled clinicians equipped with the latest research and treatment methods for childhood cancer? |
|  | Survivor | Are there streamlined Australian GP education materials and GP guidelines for survivorship management and follow-up care? |
|  | Caregiver | Why don't the nurses at the childrens hospital ED and oncology, know how to take a child's temperature properly? Inaccurately low temps result in more failed discharges which is more disruptive to the cancer kids life. |
| Health information and communication | Caregiver | [Watching my 9 month old getting a diagnosis] She relapsed at 18 months old and went through treatment for 4 months. Currently holding as much hope that she doesn’t relapse again. |
|  | Caregiver | How after all these years is there no cure for cancer? |
|  | Caregiver | Why do 1 year older get the same dose of chemotherapy as adults. |
|  | Caregiver | Was there a significant increase in the number of children diagnosed with acute lymphoblastic leukaemia post the role out of the Covid-19 vaccine? |
|  | Caregiver | How does the body experience pain and what are the ways that it can be treated? |
|  | Caregiver | Is genetic testing available to see if other family members might have a cancer gene? |
|  | Survivor | I had no idea how serious was my leukaemia. I was never told what kind it was. |
|  | Survivor | How long do I have to get to the hospital? |
|  | Health professional | How do we prepare patients and families for the journey they are about to embark upon? I.e. a new diagnosis of leukaemia. |
|  | Caregiver | Providing exhaistive list of supports that were available to child.and family when leaving hospital (even if not currently needed somifnussues arrive we know what is available) |
|  | Caregiver | As a mother I had times when I would of asked/ suggested things had I been privileged to these in a more timely manner which would have benefited the care of my daughter |
| Treatment access and research innovation and translation | Caregiver | It would have been better if we considered options outside of Chemo.for braintumours where chemo.often.dosnt work |
|  | Caregiver | Is it possible for treatment to be individualised by choice of the parent/treating team? The system seemed to be that once categorised at diagnosis that you would be not be acting in the best interest of the child for declining parts of the treatment protocol |
|  | Caregiver | What can be done at a system level to ensure cancer patients receive treatment when they need it, and not when hospital policy allows. I.e insertion or removal of Hickman/ports only being done when designated hospital lists allow, resulting in delays of effective treatment or delay in removal following the need to have one. |
|  | Caregiver | Why don’t they use donor stem cells during treatment instead of putting patients stem cells back? Apart from less chance or rejection. |
|  | Caregiver | Why is the Ewing's Sarcoma protocol so old. Why hasn't the old protocol been improved. |
|  | Caregiver | Why aren’t ports inserted sooner during treatment as peripheral administration of chemo destroys access which can be long term even after treatment |
|  | Health professional | How do we use technology to our advantage to streamline processes, rather than making them more complex every time? |
|  | Caregiver | In USA paediatric cancer patients often travel interstate to receive specialist care - should we be setting up centres of excellence in Australia for rarer types of cancer such as types of sarcoma? Would this result in better outcomes? |
|  | Caregiver | Why is Australia so far behind when it comes to drugs and treatments that are available in countries like America and England. |
|  | Caregiver | Are all worldwide treatment options available in Australia. Ie why do some families need to travel to the US for medication and treatment? |
|  | Health professional | One of the things I wonder about is how we can run clinical trials in Australia cooperatively with overseas hospitals so children do not need to travel overseas and raise hundreds of thousands of dollars for treatment. |
|  | Health professional | One of the things I wonder about is how we can get clinical trials for childhood cancers included in the Medical Treatment Overseas program |
|  | Caregiver | I have heard cancer treatment of my childs specific cancer has progressed in the research stage, but is way behind in implementing due to fund and bureaucracy. Why is this so. |
|  | Health professional | How to speed up the rate of clinical research finding treatment and cures |
|  | Caregiver | Why is there little support for development of stem cell capabilities and a national data base from the state governments? |
|  | Survivor | simpler and better ways of getting family with my treatments and progression |
|  | Caregiver | What really matters to me is being exposed to exciting and hopeful developments in treatment, such as immunotherapy. I love Australian of the Year Richard Scolyer's story and found it very uplifting (while completely understanding that this treatment is not at all transferable to my daughter's situation). It's just inspirational and connects everyone in their hopes to cure cancer. It helps us to imagine a future. |
|  | Health professional | I worry that we are in a time where everything needs to be tried, even if unlikely to have positive effect, at major expense to someone-financially, from side effect point of view and from all psychosocial aspects |
|  | Caregiver | What was likely think of what generally is long term vs med related |
|  | Caregiver | What is the best way of supporting children affected by cancer? |
|  | Caregiver | Why do we have no recent drugs that have been tested on children's cancer. |
|  | Caregiver | One of the things I will always wonder about is how much of what is learned in practice is fed back and applied as a learning to all departments  especially back to the research areas. |
|  | Caregiver | How is survivorship rates determined following death, if death was caused by cancer related not the actual cancer? |
|  | Caregiver | How do you find out about research being done on a particular type of childhood cancer |
|  | Caregiver | Very rare cancers don’t seem to attract research because of the low sample population |
|  | Caregiver | How can rhabdoid tumours be better researched. |
| Health financing | Caregiver | Who is working on our behalf to help find creative ways to incentivise research investment and drug development for Australian children with cancer? The big pharma's need commercial incentives, and there are none. Our children are dying because of red tape. |
|  | Caregiver | It would be wonderful if more money was poured into development of new treatments for childhood cancer. |
|  | Health professional | One of the things I wonder about is what incentives can we offer or legislative requirements can we put in place to ensure drug companies develop drugs for paediatric cancers. |
|  | Survivor | Are survivors entitled to concessions or rebates for the costs of medications and/or treatment of chronic long term side effects or conditions? |
|  | Caregiver | Why are corporations such as insurance companies allowed to discriminate against my child who has finished treatment and is regarded as cured but now has to pay higher premiums? |
|  | Caregiver | We shouldn’t have to rely on charities to support kids especially post treatment. What more can be done? |
|  | Health professional | How do we ensure funding goes to the most promising research and to see most needed specially for survived ones? |
|  | Caregiver | Why doesn't our government put more funding towards childhood cancers, may targeting most needed such as very rare ones or may be for personalised management activities? |
|  | Health professional | How do we ensure funding goes to the most needed research? How do we decide funding allocations to provide appreopriate care and research? |
|  | Caregiver | Why is there not funded psychologist/counselling support for once parents and kids more available. |
|  | Health professional | Why is music therapy not government funded |
|  | Caregiver | Why is the majority of current research and funding only focused on a select few cancers that already have good survival rates? Research is needed into more difficult to treat cancers or rare cancers, as these children currently have no treatment options e.g. rhabdomyosarcoma. This is an urgent matter that needs addressing |
|  | Caregiver | Can all treatments for child and adolescent cancers be funded by the government, including ones for rare cancers with no set treatment protocol/standard of care, and including overseas treatment if it is the best/only suitable option? |
|  | Caregiver | Why is there less than 2% funding allocated to children's cancer research. |
|  | Caregiver | Why is there so little money for childhood brain cancer research |
|  | Caregiver | What really matters to me is that we continue to raise awareness about childhood cancer and to continue the fundraising and research to help find a cure |
|  | Caregiver | Definitely childhood cancer and the limited funding not only in Australia but the world. |
|  | Caregiver | One of the things I wondered about was if the child’s family wasn’t in a position to fund raise or have generational wealth, did they just die. That also kept me awake at night. Why doesn’t the government help finances the overseas treatment? |
|  | Caregiver | Why doesn't the government help fund cancer research? |
| Leadership and governance | Health professional | I work in a children's hospital that cares for young people up to the age of 18 years - can see a lot of gaps that we could be doing better .. |
|  | Caregiver | What matters to me is not have ward manager push an oncology patient out while first starting treatment and to guilt trip a parent who is widowed that her child is able to walk hence for said child to be discharged despite child passing out due to zolodex while doctors still could not work out why except it had nothing to do with Oncology hence not an issue! Very poor care. |
|  | Health professional | Why do you not include GO’s more often in the care? (? Mean GPs) |
|  | Caregiver | When can parents challenge treatment paths of medical teams ? |
|  | Caregiver | Why do we sign our kids up for a treatment protocol which is hard to stray from once you have started without having done all investigative scans and tests? |
|  | Caregiver | Public awareness needs to improve about how many children actually die from cancer to support funding into research. Good news stories are always presented about survivors. If the government and public were given a realistic picture of what childhood cancer is really like, maybe there would be more funding. |
|  | Caregiver | It would have been better if we were not shut out by multiple child cancer charities because , in their words “your son died too quickly” i.e before we were engaged with their systems our son was already dead because his cancer was fast and untreatable |
|  | Caregiver | What country leads reseaching childhood cancers? |
|  | Caregiver | Can more opportunities be created for parents of children, or the children themselves where appropriate, to be involved in research and the development of future  focus of treatment? Including advisory groups, board, governance. |
|  | Caregiver | Being respected and heard for what myself as the mother knew. |
|  | Health professional | Is there a difference in priorities considered by families vs healthcare workers, and how can we better align their interests to reduce conflict? |
|  | Caregiver | I would like to see a directory of studies and or surveys such as this from a global network asking parents and survivors in depth specific questions on a number of topics to hone the types of answers we are all searching for or may be interested n in the long term and for research and development purposes. |
|  | Health professional | Hope u hear the voice of the 10-14 year olds and also siblings of all ages |
| Dimensions of care | Health professional | quality of life and patient experience |
|  | Caregiver | diagnoses and treatments in the future? |
|  | Health professional | AYA cancer care |
|  | Health professional | Psychosocial care/Early survivorship care and models of survivorship care/Pain management/Novel therapies |
|  | Survivor | Research funding and funding models |
|  | Health professional | Not sure this works, But t hink need representation of NZs |
|  | Caregiver | That every patient is different and what has work for one might not work for the other. |
|  | Caregiver | What really mattered to me was that my 19 month survived neuroblastoma. |
|  | Caregiver | I am so grateful that the doctors saved my daughters life |
|  | Caregiver | For me getting to know Dr XXX and the nursing staff was the hardest part. Once that relationship is built and knowledge of the hospital together, everything is easier. |
|  | Caregiver | It would have been better if the Covid pandemic did not mean the international bone marrow donor register could not be utilised |
|  | Caregiver | WHy do I have to raise money by donation to pay for the cost of a clinical trial? |
|  | Caregiver | How can we identify children who have a parent with cancer that need support? |
